# Modified surgical reconstruction technique for a rare isolated congenital sternal cleft: In a six -year-old child

**DOI:** 10.1016/j.amsu.2021.102280

**Published:** 2021-04-14

**Authors:** Ikram ul Haq Chaudhry, Ahsan Cheema, Chaudhry Aqeel, Ahmed Al Shaer, Fahad G. Alradei, Fatima Alquraish, Mansour Tawfeeq

**Affiliations:** Department of Surgical Specialties, Maternity and Children Hospital, Dammam, Saudi Arabia

**Keywords:** Sternum, Malformation, Cleft, Surgery, Reconstruction

## Abstract

We report the modified surgical reconstruction technique for correction for a large isolated congenital sternal cleft in 6 years old girl using a methyl methacrylate marlex mesh sandwich plate (MMS). The patient was referred to our tertiary care institution with a sizeable anterior chest wall bony defect. There was a large bulging under the skin due to protrusion of mediastinal viscera and visible cardiac pulsations with breathing. A chest x-ray and computed tomographic scan (CT) of the thorax was done to evaluate the bony defect. We reconstructed the sternal bony defect by our innovative technique using methyl methacrylate. The patient was discharged after three days for further follow up in outpatient. One year follow up patient is doing well with excellent results. Our technique is simple, cost-effective, and provides a perfect cosmetic effect for children's sternal large defects.

**Introduction:**

Sternal clefts are rare congenital malformations that result from the defective embryologic fusion of paired mesodermal bands in the midline. This rare anomaly incidence is 1:100,000 cases per live births, which constitute 1% of all congenital chest wall deformities. The first Case of the sternal cleft was reported in 1740. The hereditary sternal gap is rare, and hence sporadic cases have been reported in the medical literature. Ravitch described that the first surgical correction was reported by Lannelongue et al., in 1988., But Burton published the first successful repair in 19474. An isolated sternal cleft is a rare entity and is classified into two categories' complete and incomplete sternal gap. Early repair is recommended; otherwise, surgical correction is challenging in children as the hypo plastic sternal edges cannot be approximated primarily, and this requires prosthetic, autologous grafts or some kind of parasternal chondroplasties.

**Case presentation:**

Since birth, a six-year-old girl was referred to our tertiary care center with a large central chest wall defect. She was a full-term normal delivery with no other congenital malformations. The defect was noticed at birth and became more evident as she grew up. In addition to cosmetic concern on coughing, there was bulging under the skin and visible cardiac pulsation. On examination, there was a large gap (7cm) in the midline of the chest with sternal adages well apart, moving independently.

**Discussion:**

The sternal cleft is a congenital anomaly with less than 0.15% and is more common in the female gender. Isolated sternal cleft without any other associated abnormalities is very rare. It has been reported as a part of defined syndromes like PENTALOGY OF CANTRELL, VACTREL, DANDY WALKER, and PHASE (Posterior fossa brain malformation, hemangioma, arterial lesions, cardiac abnormalities, and eye abnormality).6 Embryo logically sternum originate from the somatic layer of lateral mesodermal plates as bilateral bands. They fuse in the midline by the 10th week to constitute a cartilaginous framework of manubrium, sternum, and xiphoid process. Failure of this fusion can lead to a partial or complete sternal cleft.7 Etiology of this disease is unknown; however, it has been linked with riboflavin or methyl-cobalamin deficiency, high alcohol intake during pregnancy.

**Conclusion:**

in conclusion, our improvised reconstruction technique for large sternal cleft in children has several advantages. There is no need to do extensive chordotomies or using bone grafts. Less complicated procedure Provides more rigid frame for protection of thoracic structures and better chest wall stability. Hospital stay is minimal and is very cost-effective. The child's future growth is not affected as ribs and costal cartilages are left intact in this technique. There is no chance of displacement or excursion of the MMS plate. In female patients, this provides better cosmoses as there is no need to mobilize the pectoralis significant muscles flaps for coverage. The geometry of the rib cage is well preserved.

## Highlights

• A six years old girl presented with wide sternal gap, sternal edges moving apart paradoxically.
• Mediastinal viscera were bulging under the skin and visible cardia pulsations.
• A computed tomographic scan (CT)of the chest 3 D reconstruction revealed a large sternal cleft.
• The sternal cleft was surgical repaired with our Modifies reconstruction technique with excellent results.
• Our technique provides excellent chest wall stability and very good cosmoses.

## 1 Background

Sternal clefts are rare congenital malformations that result from the defective embryologic fusion of paired mesodermal bands in the midline. This rare anomaly incidence is 1:100,000 cases per live births, which constitute 1% of all congenital chest wall deformities [1,2]. The first Case of the sternal cleft was reported in 1740. The hereditary sternal gap is rare, and hence sporadic cases have been reported in the medical literature. Ravitch described that the first surgical correction was reported by Lannelongue et al., in 1988. [3]^,^ But Burton published the first successful repair in 1947 [4]. An isolated sternal cleft is a rare entity and is classified into two categories' complete and incomplete sternal gap. Early repair is recommended; otherwise, surgical correction is challenging in children as the hypoplastic sternal edges cannot be approximated primarily, and this requires prosthetic, autologous grafts or some kind of parasternal chondroplasties [5].This case has been reported in line with SCARE criteria [6].

## 2 Case report

**Fig. 1:**
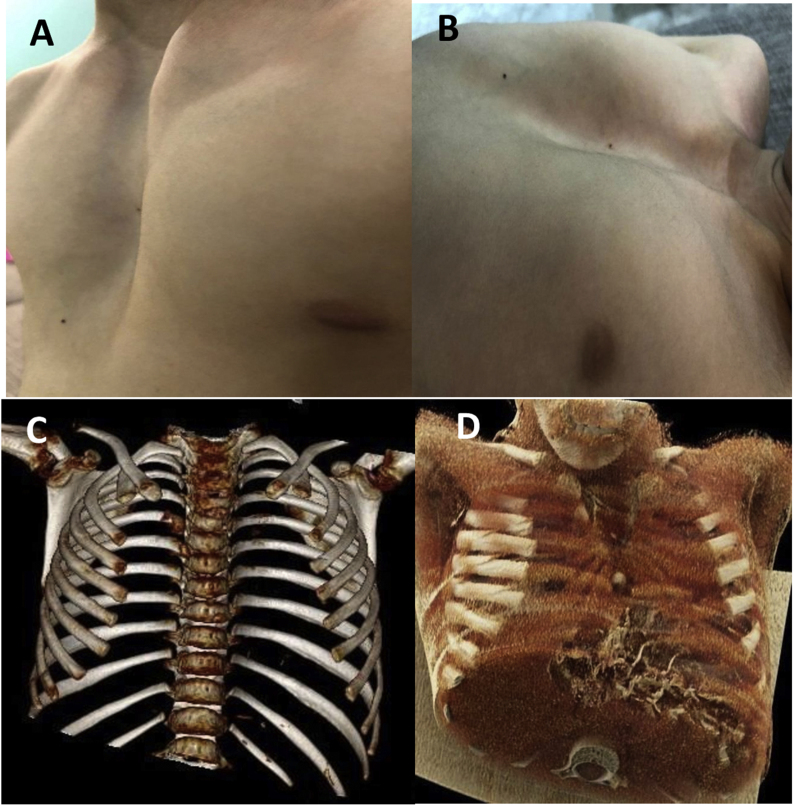
A. Patient's photograph in standing position showing wide sternal gap. B. Patient's photograph in supine position showing wide sternal gap. C & D. 3D CT scan of Thorax showing congenitally absent sternum.

**Fig. 2:**
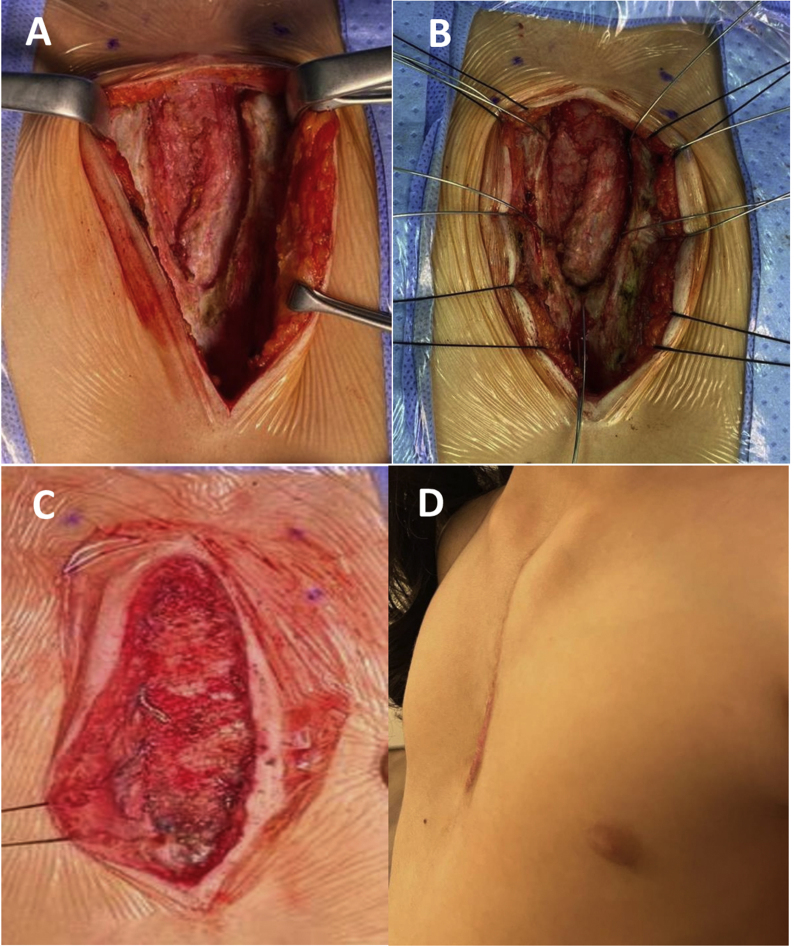
A. Per operative photograph showing V shaped sternal defect. B. a photograph showing sternal wires for fixation. C. Reconstructive plate fixed in place. D. Post-operative photograph of the patient in standing position.

Since birth, a six-year-old girl was referred to our tertiary care center with a large central chest wall defect. She was a full-term normal delivery with no other congenital malformations. The defect was noticed at birth and became more evident as she grew up. In addition to cosmetic concern on coughing, there was bulging under the skin and visible cardiac pulsation. On examination, there was a large gap (7cm) in the midline of the chest with sternal adages well apart, moving independently. Fig. 1: A &B. Necessary blood investigations liver and renal panel were normal. Chest x-ray showed that the sternum body had not ossified, and the medial ends of the sternum were far away from the midline The lung fields were clear. CT of thorax with the 3-D scan of the thorax showed a large sternal cleft Fig. 1: C & D. They already had an opinion from the surgeon of another institute who explained the defect would be covered by bilateral pectoralis major muscle flaps and bone graft. Parents were concerned about the cosmoses of their girl. A family meeting was arranged to discuss the reconstruction of the defect, and our surgical technique and material to be used for reconstruction were explained, and they consented to the procedure and photographs. Patient was placed in a supine position under general anesthesia. She received prophylaxis antibiotic, cephalosporin. A midline incision was made from the sternal notch to the xiphoid process. Sternal edges were exposed by raising the bilateral muscle flaps, and pericardium and pleura were preserved and dissected free of sternal edges. Fig. 2 A The defect was measured, and the MMS plate was made and molded according to the defect's shape and neosternal notch was created. Holes were drilled in the sternal edges and correspondingly in the MMS plate. Wires (5 mm) were passed through the bone, and the MMS plate was fixed by twisting the wires, and the prolene mesh was sutured with 2–0 prolene to the surrounding tissues. Fig. 2: B & C. The anterior chest wall wound was closed by approximating the soft tissues and pectoralis major muscles. The skin was closed without the use of a myocutaneous flap.

The patient was extubated on the table and transferred to a high dependency unit for overnight observation. The patient had an uneventful post-operative recovery and was discharged home for follow up in outpatient clinic. The wound was nicely healed without any residual defect. Fig. 2: D.

**Fig. 3:**
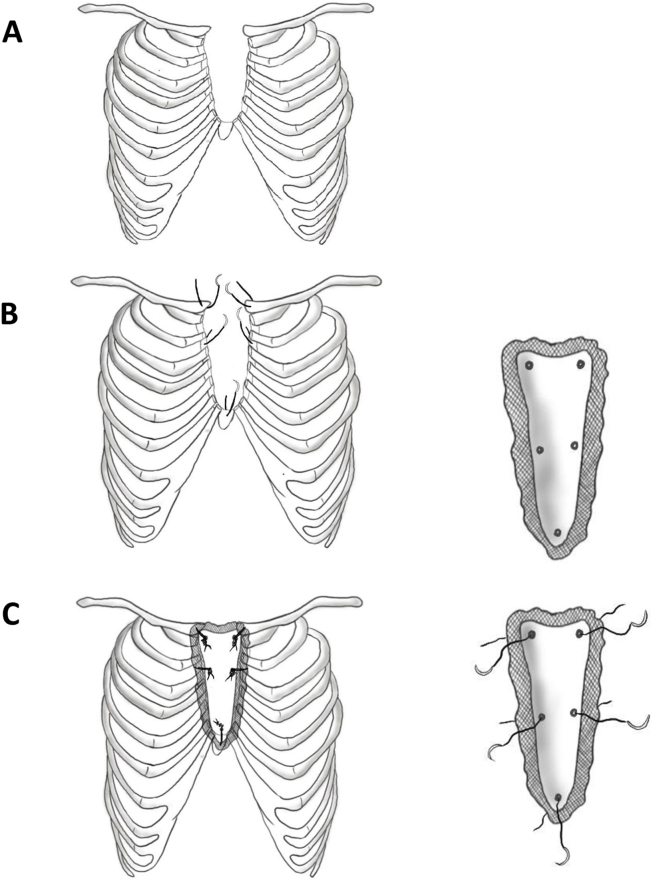
A. Illustrative drawings of the congenital sternal defect. B. Anchoring sternal wires in place, with the (Methyl Methacrylate Marlex mesh) reconstruction plate shown separately with corresponding holes. C. Final sketch showing reconstructive plate fixed in the defect and the plate before fixation shown separately with wires passed through the plate prior to fixation.

Operative steps are shown in illustrated drawings Fig. 3: A, B & C.

## 3 Discussion

The sternal cleft is a congenital anomaly with less than 0.15% and is more common in the female gender. Isolated sternal cleft without any other associated abnormalities is very rare. It has been reported as apart of defined syndromes like PENTALOGY OF CANTRELL, VACTREL, DANDY WALKER, and PHASE (Posterior fossa brain malformation, hemangioma, arterial lesions, cardiac abnormalities, and eye abnormality [7]. Embryologically sternum originate from the somatic layer of lateral mesodermal plates as bilateral bands. They fuse in the midline by the 10th week to constitute a cartilaginous framework of manubrium, sternum, and xiphoid process. Failure of this fusion can lead to a partial or complete sternal cleft [8]. Etiology of this disease is unknown; however, it has been linked with riboflavin or methyl-cobalamin deficiency, high alcohol intake during pregnancy.

Sternal cleft due to disruption of Hoxb −4 gene has been reported in the mouse by Ramirez-Solis et al. [9]^.^ The sternal clefts are classified as complete or incomplete, which are further subdivided into superior and inferior. Morphologically they are described as U or V-shaped as the u shape extends to the 4th costal cartilage while the V shape extends to the xiphoid [10]. Hersh et al., in 1985 classified sternal cleft into four types. Type 1 isolated sternal cleft without any associated anomalies, Type II is associated with vascular dysplasia, Type III is with ectopia cordis, Type IV with Cantrell's pentalogy [11]. Diagnosis is generally is on physical examination is evident as the sternum is absent, so there is a gap at the site of the sternum, and cardiac pulsations are visible under the skin. And hypoplastic sternal edges move separately. On deep breathing and coughing or Valsalva maneuver, alternate protrusion and retraction movements are leading to bulging of lungs and mediastinal viscera. Due to the lack of bony protection heart, great vessels, and lungs are vulnerable to external trauma [12]. Due to the thoracic viscera's paradoxical movements during respiration, the incidence of respiratory infection is more in such children because of inability to increase intrathoracic pressure, which leads to decreased cough reflex and lung aeration. The patients with large sternal defects are prone to cardiac arrhythmia and commotio cardis. The best diagnostic modality is computed tomographic of thorax with 3-D reconstruction pictures, which are helpful to evaluate the type and magnitude of the sternal defect and select an appropriate material for reconstruction [13,14].Surgery is mandatory for such congenital disability. Ideally the sternal cleft should be corrected in the neonatal period because primary closure is easy due to good chest wall flexibility, and underlying compression is minimal [15,16]. There are several reconstruction surgical techniques reported in the medical literature. First primary closure was reported in 1949 by Maier and Bortone in six weeks old baby [17]. Ballouhey et al. used autogenous material a double ostechondroplasty flaps for reconstruction in infants [[18], [19]]. In older children, surgical repair is challenging, and many surgical reconstruction techniques have been reported in the medical literature. Sliding chordotomy by (Sabiston), cartilage resection, and primary. Closure by **s**ally et al. [20,21].

**Fig. 4:**
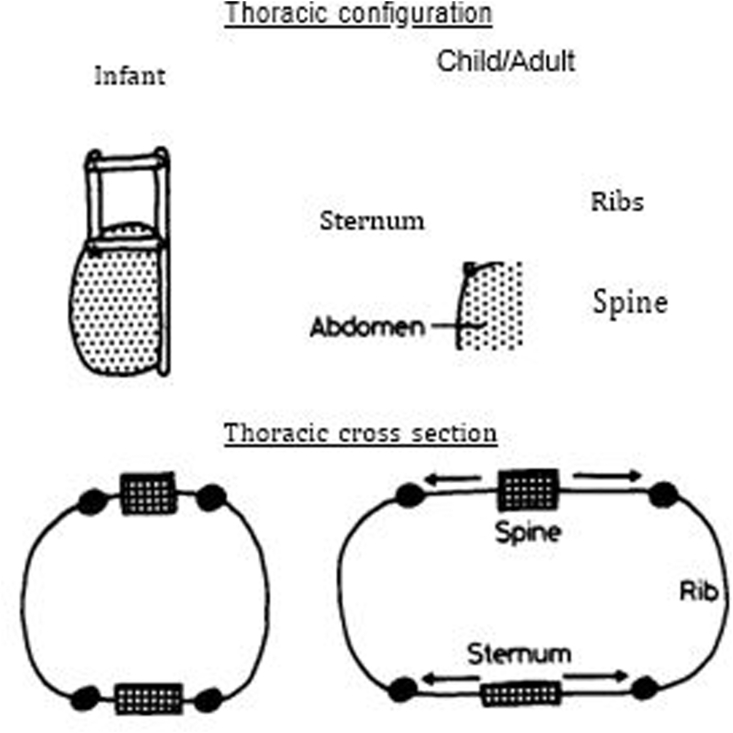
Changes in rib cage geometry in childhood.

Pectoralis major muscle bilateral muscle flaps, cartilage graft bridging, bone grafts, prosthetic materials, titanium plates, marlex mesh, Teflon, and silicone prosthesis has been used for reconstruction [[22], [23], [24], [25], [26]]. The surgical technique should be designed according to the age and size of the defect estimated by studying the thorax's 3-D CT scan of the thorax. Large defects required prosthetic material for closure. We used methyl methacrylate cement with prolene mesh for reconstruction. The future growth of chest wall bony structures should not be affected, and the geometry of the chest should be preserved. Geometrical changes in the rib cage during childhood are reported by Openshaw et al., in 1984 [27].Methyl methacrylate cement sandwich in prolene mesh molded according to the geometry of chest wall defect is one of the best materials to provide optimal reconstruction with maximum stability. The only drawback is wound complications and tilting or excursion of the plate. With our improvised technique, there is no chance of MMS plate tilting or excursion. The Changes in rib cage geometry during childhood reported by Penshaw et al. [28] Fig. 4.

In conclusion, our improvised reconstruction technique for large sternal cleft in children has several advantages. There is no need to do extensive chordotomies or using bone grafts. Less complicated procedure Provides more rigid frame for protection of thoracic structures and better chest wall stability. Hospital stay is minimal and is very cost-effective. The child's future growth is not affected as ribs and costal cartilages are left intact in this technique. There is no chance of displacement or excursion of the MMS plate. In female patients, this provides better cosmoses as there is no need to mobilize the pectoralis significant muscles flaps for coverage. The geometry of the rib cage is well preserved.

## Sources of funding

No source of funding.

## Trial registry number

1. Name of the registry: Research registry
2. Unique Identifying number or registration ID: 6710
3. Hyperlink to your specific registration (must be publicly accessible and will be checked): http://www.researchregistry.com/browse-the-registry#home/

## Declaration of competing interest

No conflict of interest and there was no source of funding or financial assistance in this Case.

## Ethical approval

IRB Approval.

## Consent

Yes written consent obtained from the father of the child.

## Author contribution

IUC, Operating surgeon drafting the article, Critical revision and final approval of the article. (Corresponding author).

AC, wrote structured abstract.

CA wrote abstract.

AAA highlights.

FA searched references.

ZA illustrative drawings.

MT wrote the part of discussion and review.

## Guarantor

Ikram ul haq Chaudhry.

## Provenance and peer review

Not commissioned, externally peer reviewed.
